# Body weight loss as a prognostic and predictive factor in previously treated patients with metastatic gastric cancer: post hoc analyses of the randomized phase III TAGS trial

**DOI:** 10.1007/s10120-023-01393-2

**Published:** 2023-04-28

**Authors:** Michele Ghidini, Howard Hochster, Toshihiko Doi, Eric Van Cutsem, Lukas Makris, Osamu Takahashi, Karim A. Benhadji, Wasat Mansoor

**Affiliations:** 1 Oncology Division, Azienda Socio Sanitaria Territoriale di Cremona, Azienda Ospedaliera di Cremona, Viale Concordia 1, 26100 Cremona, Italy; 2 Gastrointestinal Oncology, Rutgers Cancer Institute, New Brunswick, NJ USA; 3 Department of Experimental Therapeutics, National Cancer Center Hospital East, Chiba, Japan; 4 Digestive Oncology, University Hospitals Gasthuisberg, Leuvain, Belgium; 5 Stathmi, Inc, New Hope, PA USA; 6 Taiho Oncology, Inc., Princeton, NJ USA; 7 Department of Medical Oncology, The Christie NHS Foundation Trust, Manchester, UK

**Keywords:** Gastric cancer; gastroesophageal junction cancer, Trifluridine tipiracil, Body weight loss, Prognostic factor

## Abstract

**Background:**

Body weight loss (BWL) is a negative prognostic factor in metastatic gastric or gastroesophageal junction cancer (mGC/GEJC). In the phase III TAGS study, trifluridine/tipiracil improved survival versus placebo in third- or later-line mGC/GEJC. These retrospective analyses examined the association of early BWL with survival outcomes in TAGS.

**Methods:**

Efficacy and safety were assessed in patients who experienced < 3% or ≥ 3% BWL from treatment start until day 1 of cycle 2 (early BWL). The effect of early BWL on overall survival (OS) was assessed by univariate and multivariate analyses.

**Results:**

Body weight data were available for 451 of 507 (89%) patients in TAGS. In the trifluridine/tipiracil and placebo arms, respectively, 74% (224/304) and 65% (95/147) experienced < 3% BWL, whereas 26% (80/304) and 35% (52/147) experienced ≥ 3% BWL at cycle 1 end. Median OS was longer in < 3% BWL versus ≥ 3% BWL subgroups (6.5 vs 4.9 months for trifluridine/tipiracil; 6.0 vs 2.5 months for placebo). In univariate analyses, an unadjusted HR of 0.58 (95% CI, 0.46–0.73) for the < 3% vs ≥ 3% BWL subgroup indicated a strong prognostic effect of early BWL. Multivariate analyses confirmed early BWL as both prognostic (*P* < 0.0001) and predictive (interaction *P* = 0.0003) for OS. Similar results were obtained for progression-free survival. Any-cause grade ≥ 3 adverse events were reported in 77% and 82% of trifluridine/tipiracil-treated and 45% and 67% of placebo-treated patients with < 3% and ≥ 3% BWL, respectively.

**Conclusions:**

In TAGS, early BWL was a strong negative prognostic factor for OS in patients with mGC/GEJC receiving third- or later-line treatment.

**Supplementary Information:**

The online version contains supplementary material available at 10.1007/s10120-023-01393-2.

## Introduction

Gastric cancer (GC) is the third most common cause of cancer-related death worldwide [1, 2]. While surgical resection is considered one of the only curative treatments, a majority of radically resected patients experience recurrent disease [2–4]. Chemotherapy improves survival outcomes in patients with advanced, unresectable or metastatic GC (mGC), yet median overall survival (OS) is limited to 12 months following therapy with combination chemotherapy regimens [4]. The recommended treatment for advanced, metastatic disease in the first-line setting includes a platinum and fluoropyrimidine doublet (with trastuzumab for human epidermal growth factor receptor-2 [HER2] disease) [2]. More recently, the combination of immune checkpoint inhibitors (nivolumab or pembrolizumab) with chemotherapy or trastuzumab or both have shown efficacy in the first-line setting [5–7]. Second-line treatment options include taxanes, irinotecan, and the vascular endothelial growth factor receptor-2 inhibitor ramucirumab [2], along with trastuzumab deruxtecan for patients with HER2-positive GC previously treated with trastuzumab [8].

In the third- or later-line setting, trifluridine and tipiracil (FTD/TPI; TAS-102), comprising trifluridine, a thymidine analog, and tipiracil, a thymidine phosphorylase inhibitor [9], has shown clinical benefit. In the randomized, double-blind, placebo-controlled, phase III TAGS trial, FTD/TPI improved survival versus placebo (median OS, 5.7 vs 3.6 months; hazard ratio [HR], 0.69; 95% confidence interval [CI], 0.56–0.85; *p* = 0.00058) in patients with mGC or gastroesophageal junction cancer (mGC/GEJC) who had received ≥ 2 prior chemotherapy regimens [10]. FTD/TPI showed a manageable safety profile, with hematologic and gastrointestinal adverse events (AEs) being most commonly observed [10].

As response to systemic therapy varies from individual to individual, it is important to identify patients who will benefit most from second- or third-line chemotherapy. In this context, understanding prognostic factors for survival and quality of life may help clinicians optimize and individualize treatment, hopefully providing an opportunity to intervene and change the course of the disease [11–13]. Nutritional status has been correlated with cancer mortality, and body weight loss (BWL) was shown to be superior to other indices of nutritional status as a prognostic indicator of survival and quality of life [14]. BWL has been explored as a prognostic factor in curative, first-, and second-line settings in mGC/GEJC [14–21]. An overall BWL of ≥ 10% within 3 months of treatment was shown to be a negative prognostic indicator for survival and quality of life [22]. In a pooled analysis of 2 randomized clinical trials of patients undergoing second-line treatment for advanced GC, a ≥ 10% weight loss within 3 months of the study baseline evaluation was associated with worse OS compared with a < 10% weight loss [22]. Early BWL, which occurs in the first cycle of systemic treatment, was also shown to have prognostic and predictive associations with outcomes [18, 23]. In a retrospective analysis of 719 patients with advanced GC who were receiving palliative chemotherapy, > 3% BWL over the first month of palliative chemotherapy was predictive of unfavorable survival outcomes [23]. A post hoc analysis of 3 phase III studies of ramucirumab in mGC (RAINBOW, REGARD and RAINFALL) also found that weight loss of ≥ 3% in the first cycle of treatment (or early BWL) was a negative prognostic factor for OS in patients treated in first- and second-line settings [18]. However, such data are lacking in patients with mGC/GEJC being treated in the third- and later-line settings.

Here, we report results from a retrospective, post hoc analysis of the phase III TAGS trial, in which we examined the association of early BWL with survival outcomes and safety in patients with mGC/GEJC treated with FTD/TPI or placebo in the third- or later-line setting.

## Methods

### Patients and study design

The study design for the randomized, double-blind, placebo-controlled, phase III TAGS study (NCT02500043) has been reported previously [10]. Briefly, eligible patients (with Eastern Cooperative Oncology Group performance status [ECOG PS] of 0 or 1) had histologically confirmed, non-resectable, metastatic gastric adenocarcinoma (including adenocarcinoma of the gastroesophageal junction), had received at least 2 previous chemotherapy regimens, and had experienced radiological disease progression or were unable to tolerate the most recent therapy. The study was conducted at 110 academic hospitals in 17 countries. Patients were randomized in a 2:1 ratio to receive either oral FTD/TPI (35 mg/m^2^ twice daily on days 1–5 and days 8–12 of each 28-day cycle) plus best supportive care (BSC) or placebo plus BSC. Randomization was stratified by region (Japan vs rest of the world), ECOG PS (0 vs 1), and prior ramucirumab treatment (yes vs no). Treatment continued until disease progression, intolerance, or patient withdrawal, or completion of the primary endpoint, whichever occurred first. The data cutoff for this analysis was March 31, 2018.

TAGS was designed and conducted in accordance with the Declaration of Helsinki and ethical principles of good clinical practice and local regulations. All protocols and amendments were approved by the institutional review boards and ethics committees prior to study initiation. All patients provided written informed consent prior to study participation.

The primary endpoint of the study was OS. Key secondary endpoints were progression-free survival (PFS) and safety and tolerability.

### Study assessments

Tumor assessments by computer tomography of the chest and abdomen were done within 28 days before cycle 1, and every 8 weeks during study treatment and evaluated by the investigator according to Response Evaluation Criteria for Solid Tumors version 1.1 (RECIST v1.1). Safety was assessed throughout the study, and AEs were graded according to National Cancer Institute’s Common Terminology Criteria version 4.03. Weight was measured in the week before the start of treatment, within 24 h of the first day of each cycle starting with cycle 2, and at end of treatment and safety follow-up visits.

### Early BWL subgroup analysis

This retrospective, post hoc analysis examined the effect of BWL on survival and safety outcomes in patients from the TAGS study. Patients with body weight data available at the end of cycle 1 from the TAGS population were categorized into early BWL subgroups (< 3% or ≥ 3% BWL) based on weight change from start of treatment until day 1 of cycle 2. The 3% threshold was selected as a cutoff based on a previous analysis showing an association with negative survival outcomes in patients with mGC [18, 23]. By definition, all patients with available body weight data at the end of cycle 1 had received at least 1 dose of study drug*.* Efficacy and safety were compared between BWL subgroups within each treatment arm because there was an imbalance in the distribution of patients between the treatment arms in the BWL subgroups.

### Statistical analyses

Statistical considerations for the overall study have been previously reported. The analyses in BWL subgroups were exploratory and not powered for formal hypothesis testing, and therefore no *p-*values were reported for any comparisons between the subgroups. Baseline demographics, disease characteristics, and prior treatments were reported descriptively. Safety was evaluated using descriptive statistics within treatment and BWL subgroups. Kaplan–Meier estimates were used to calculate median OS or PFS within BWL subgroups.

The effect of early BWL on OS was assessed by univariate and multivariate analyses performed using Cox’s proportional hazards model. For univariate analyses, weight loss change at the end of cycle 1 (< 3% vs ≥ 3% BWL) was used as the only covariate. The multivariate analyses adjusted for baseline prognostic factors identified in the original intention-to-treat (ITT) analysis. These factors included the stratification factors (region, ECOG status, prior ramucirumab), age (< 65 vs ≥ 65 years), number of prior regimens (≤ 2 vs ≥ 3), number of metastatic sites (1–2 vs ≥ 3), histology subtype (intestinal vs diffused, mixed or unknown) and HER2 status at baseline (negative vs positive/not done) [10]. A test was conducted for interaction with treatment.

## Results

### Patient demographics and disposition

**Fig. 1:**
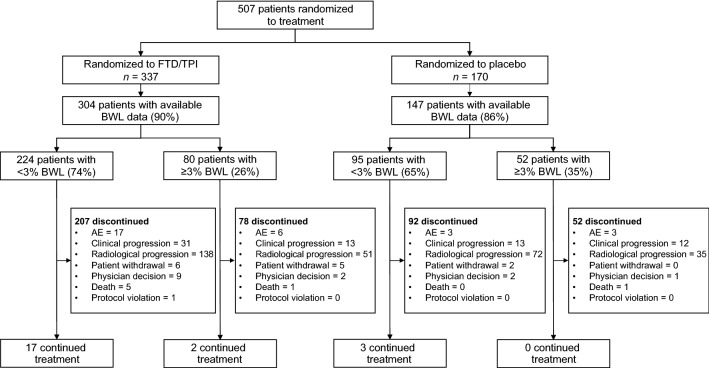
Patient disposition by BWL subgroup. *AE* adverse event, *BWL* body weight loss, *FTD/TPI* trifluridine/tipiracil

In the TAGS trial, a total of 507 patients were enrolled and randomized (337 to FTD/TPI and 170 to placebo) between February 24, 2016, and January 5, 2018 [10]. Among these 507 patients, body weight data were available for 451 patients (89%; 304/337, FTD/TPI; 147/170, placebo) at the end of cycle 1 of treatment. The majority of patients (319/451; 71%) had < 3% BWL, of whom 224/319 (70%) received FTD/TPI and 95/319 (30%) received placebo. Overall, 132/451 patients (30%) experienced ≥ 3% BWL at the end of cycle 1, of whom 80/132 (60%) received FTD/TPI and 52/132 (39%) received placebo (Fig. 1). Because of this imbalance in distribution of patients between the treatment arms (the placebo arm had a numerically higher proportion of patients with ≥ 3% BWL than the FTD/TPI arm), data were compared between BWL subgroups within each treatment arm.

Patient baseline demographics, disease characteristics and previous treatments were generally similar between patients with < 3% or ≥ 3% BWL (Table 1) with a few exceptions. Compared with patients with < 3% BWL, a numerically higher percentage of patients with ≥ 3% BWL had an ECOG PS of 1 (70% [93/132] vs 56% [179/319]), negative HER-2 status (69% [91/132] vs 56% [179/319]) and ≥ 3 metastatic sites (64% [85/132] vs 50% [161/319]). In addition, 24% (32/132) of patients in the ≥ 3% BWL group had ascites compared to 20% (64/319) in the < 3% BWL group.

**Table 1:** Baseline demographics, disease characteristics, and prior treatment of patients with < 3% or ≥ 3% BWL

Characteristic	Patients with < 3% BWL		Patients with ≥ 3% BWL	
	FTD/TPI (*n* = 224)	Placebo (*n* = 95)	FTD/TPI (*n* = 80)	Placebo (*n* = 52)
Age				
Median (range), years	64 (24–89)	64 (32–82)	61 (27–82)	58 (39–76)
Age category, *n* (%)				
< 65 years	117 (52)	49 (52)	49 (61)	37 (71)
≥ 65 years	107 (48)	46 (48)	31 (39)	15 (29)
Body mass index Median (range), m/kg^2^	23 (15–38)	24 (14–36)	23 (15–35)	23 (15–41)
Sex, *n* (%)				
Male	166 (74)	66 (69)	60 (75)	36 (69)
Female	58 (26)	29 (31)	20 (25)	16 (31)
Ethnicity, *n* (%)^a^				
Caucasian/White	162 (72)	68 (72)	56 (70)	31 (60)
Asian	34 (15)	16 (17)	15 (19)	9 (17)
Other/not collected	28 (13)	11 (12)	9 (11)	12 (23)
Region, *n* (%)				
Europe	181 (81)	76 (80)	60 (75)	41 (79)
Japan	31 (14)	16 (17)	13 (16)	9 (17)
USA	12 (5)	3 (3)	7 (9)	2 (4)
ECOG PS, *n* (%)				
0	94 (42)	46 (48)	21 (26)	18 (35)
1	130 (58)	49 (52)	59 (74)	34 (65)
Primary cancer type, %				
Gastric	160 (71)	68 (72)	56 (70)	35 (67)
Gastroesophageal junction	64 (29)	27 (28)	24 (30)	15 (29)
Both	0	0	0	2 (4)
Measurable disease, *n* (%)	204 (91)	88 (93)	71 (89)	42 (81)
Histology subtype, *n* (%)				
Intestinal	74 (33)	30 (32)	21 (26)	17 (33)
Diffuse	33 (15)	10 (11)	16 (20)	7 (13)
Mixed	9 (4)	4 (4)	3 (4)	2 (4)
Unknown	88 (39)	36 (38)	30 (38)	23 (44)
Not available	20 (9)	15 (16)	10 (12)	3 (6)
HER2 status, *n* (%)				
Positive	48 (21)	18 (19)	13 (16)	7 (13)
Negative	125 (56)	54 (57)	56 (70)	35 (67)
Unavailable	50 (22)	23 (24)	11 (14)	10 (19)
Number of metastatic sites, *n* (%)				
1–2	110 (49)	48 (51)	30 (38)	17 (33)
≥ 3	114 (51)	47 (49)	50 (62)	35 (67)
Peritoneal metastases, *n* (%)	56 (25)	27 (28)	20 (25)	15 (29)
Previous gastrectomy, *n* (%)	105 (47)	39 (41)	32 (40)	25 (48)
Prior radiotherapy, *n* (%)	42 (19)	14 (15)	23 (29)	9 (17)
Number of prior regimens, *n* (%)				
2	86 (38)	35 (37)	30 (38)	23 (44)
3	83 (37)	36 (38)	30 (38)	14 (27)
≥ 4	55 (25)	24 (25)	20 (25)	15 (29)
Number of prior regimens for metastatic cancer, *n* (%)				
1	9 (4)	1 (1)	4 (5)	1 (2)
2	106 (47)	41 (43)	31 (39)	28 (54)
3	71 (32)	37 (39)	32 (40)	15 (29)
≥ 4	38 (17)	16 (17)	13 (16)	8 (15)
Prior systemic regimens for metastatic cancer, *n* (%)^b^				
Fluoropyrimidines^b^	217 (97)	92 (97)	78 (98)	49 (94)
Platinum-containing compounds^c^	210 (94)	92 (97)	74 (92)	49 (94)
Taxanes^d^	200 (89)	79 (83)	76 (95)	45 (87)
Irinotecan^e^	126 (56)	58 (61)	41 (51)	26 (50)
HER2-inhibitors^f^	41 (18)	16 (17)	12 (15)	6 (12)
Immunotherapy (anti-PD1/PD-L1 agents)	19 (8)	2 (2)	4 (5)	2 (4)
Other	59 (26)	22 (23)	11 (14)	12 (23)

*ECOG PS* eastern cooperative oncology group performance status, *FTD/TPI* trifluridine/tipiracil, *HER2* human epidermal growth factor receptor 2, *PD1* programmed cell death protein 1, *PD-L1* programmed death-ligand 1, *SD* standard deviation

^b^Patients may have identified as more than one race or ethnicity. ^b^Patients could have received more than one category of treatment; ^b^Included 5-fluorouracil, capecitabine, doxifluridine, S-1, tegafur and tegafur/uracil (UFT), and agents collected as 'other' and later re-mapped to ‘fluoropyrimidines’; ^c^Included oxaliplatin, cisplatin, carboplatin and agents that collected as 'other' and later re-mapped to ‘platinum-containing compounds’; ^d^Included docetaxel, paclitaxel, nab-paclitaxel (abraxane), and agents that collected as 'other' and later re-mapped to ‘taxanes’; ^e^Included irinotecan and CPT-11, and agents that collected as 'other' and later re-mapped to ‘irinotecan’; ^f^Included trastuzumab, pertuzumab and T-DM1

The proportion of patients who discontinued FTD/TPI for any reason was 92% (207/224) in the < 3% BWL subgroup and 98% (78/80) in the ≥ 3% BWL subgroup. Among patients receiving placebo, 97% (92/95) in the < 3% BWL subgroup and 100% (52/52) in the ≥ 3% BWL subgroup discontinued treatment (Fig. 1; Table S1). In both subgroups, the most common reason for treatment discontinuation was clinical or radiological progression, observed in 75% (169/224) and 80% (64/80) of FTD/TPI-treated patients with < 3% and ≥ 3% BWL, respectively, and 89% (85/95) and 90% (47/52) of placebo-treated patients with < 3% and ≥ 3% BWL, respectively.

### Efficacy by BWL

**Fig. 2:**
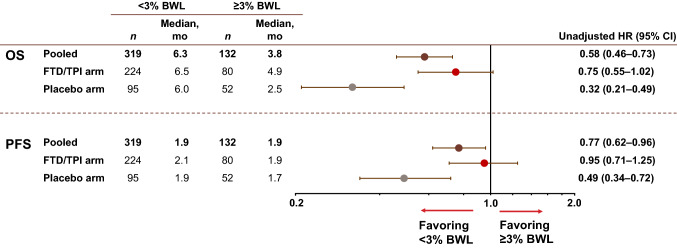
OS and PFS by BWL subgroup. Figure shows a forest plot of OS and PFS HRs in the pooled patient population, FTD/TPI and placebo arms. *BWL* body weight loss, *CI* confidence interval, *HR* hazard ratio, *mo* months, *OS* overall survival, *PFS* progression-free survival. Median values and hazard ratios calculated using Kaplan–Meier estimates

**Fig. 3:**
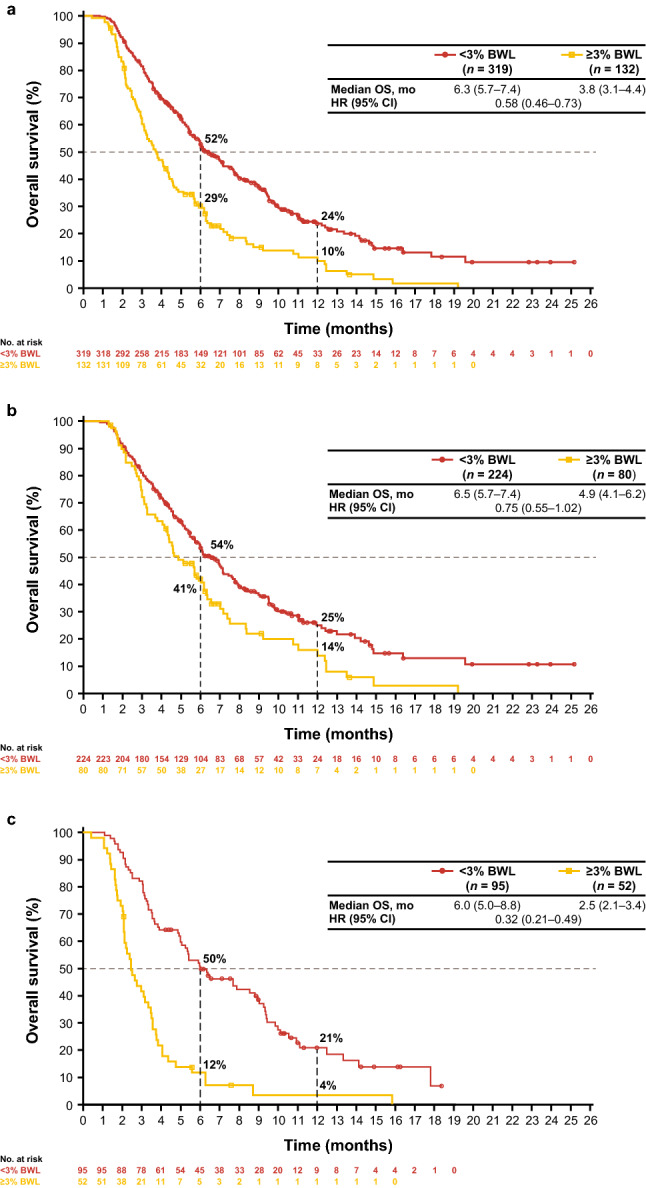
OS in patients with < 3% or ≥ 3% BWL. Kaplan–Meier plots of OS by < 3% or ≥ 3% BWL in **a** The pooled patient population, **b** FTD/TPI arm and **c** Placebo arm. Kaplan–Meier estimates were used to calculate OS, 95% CI being produced via the methodology of Brookmeyer and Crowley. *BWL* body weight loss, *CI* confidence interval, *HR* hazard ratio; *OS* overall survival

In this trial, patients with a BWL of < 3% at the end of cycle 1 experienced longer median OS than patients with ≥ 3% BWL, both in the pooled population and across treatment arms (Figs. 2 and 3). In the pooled population and FTD/TPI arms respectively, median OS was 6.3 and 6.5 months in the < 3% BWL group and 3.8 and 4.9 months in the ≥ 3% BWL group. The effect of early BWL on OS was most pronounced in the placebo group in which patients with < 3% BWL had a median OS of 6.0 months compared with 2.5 months in those with ≥ 3% BWL.

These findings are supported by analyses from the univariate Cox proportional hazards model, which indicated a strong prognostic effect of early BWL on OS. The unadjusted HR in the pooled ITT population for < 3% versus ≥ 3% BWL was 0.58 (95% CI, 0.46–0.73). Within the FTD/TPI treatment arm and the placebo arms, the unadjusted HRs were 0.75 and 0.32, respectively. Multivariate analyses were consistent with univariate analyses and indicated that BWL at the end of cycle 1 was both a prognostic (*p* < 0.0001) and predictive factor (interaction *p* = 0.0003) for OS (Table 2).

**Table 2:** Multivariate analysis of OS in patients with < 3% or ≥ 3% BWL

Variable	HR^a^ (95% CI)	*p* value^b^	Interaction *p *value^c^
Treatment (FTD/TPI vs placebo)	0.66 (0.52 − 0.83)	0.0003	NA
Region (Japan vs rest of world)		0.93	0.94
ECOG performance status (0 vs 1)		< 0.0001	0.80
Prior ramucirumab (yes vs no)		0.93	0.54
Age (< 65 vs ≥ 65 years)		0.0002	0.20
Number of prior regimens (≤ 2 vs ≥ 3)		0.053	0.53
Number of metastatic sites (1 − 2 vs ≥ 3)		0.0021	0.87
Histology subtype (intestinal vs diffuse, mixed or unknown)		0.15	0.69
Baseline HER2 status (negative vs positive or not done)		0.18	0.11
BWL at end of cycle 1 (< 3% vs ≥ 3%)		< 0.0001	0.0003

*BWL* body weight loss. *CI* confidence interval. *ECOG* Eastern Cooperative Oncology Group. *FTD/TPI* trifluridine/tipiracil. *HER2* human epidermal growth factor 2. *HR* hazard ratio. *NA* not applicable. *OS* overall survival

^a^Derived using Cox regression that included all factors shown

^b^Derived using the Wald chi-square test

^c^*p* value for interaction with treatment from the complete model plus 2-way interaction with BWL at the end of cycle 1

**Fig. 4:**
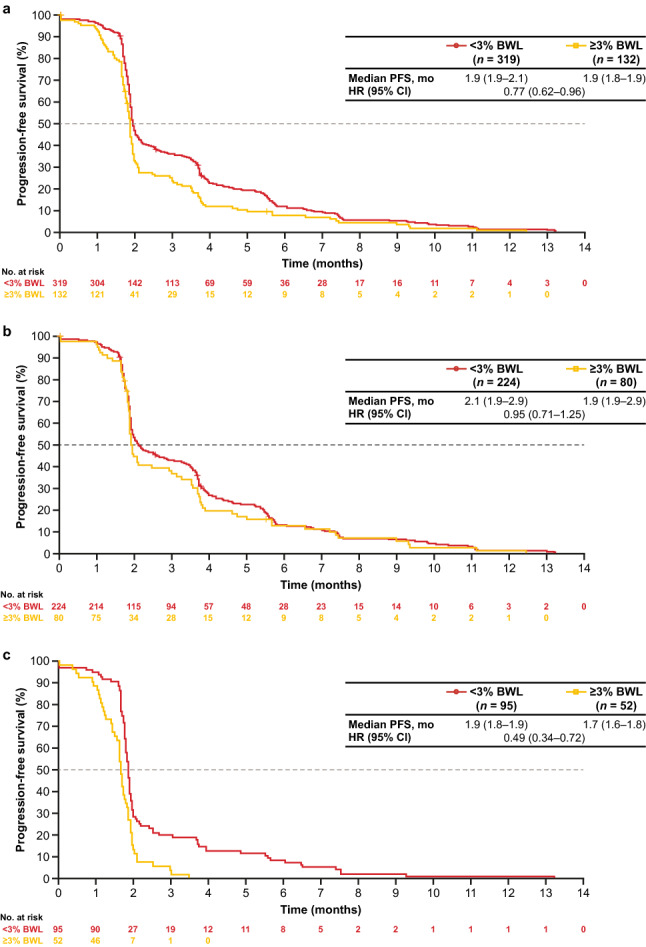
PFS in patients with < 3% or ≥ 3% BWL. Kaplan–Meier plots of PFS by < 3% or ≥ 3% BWL in **a** The pooled patient population, **b** FTD/TPI arm and **c** Placebo arm. Kaplan–Meier estimates were used to calculate PFS, 95% CI being produced via the methodology of Brookmeyer and Crowley. *BWL* body weight loss, *CI* confidence interval, *HR* hazard ratio; *PFS* progression-free survival

Similar trends were observed with PFS (Figs. 2 and 4) and OS. Longer PFS values were observed in patients with < 3% BWL than in those with ≥ 3% BWL both in the pooled population and across treatment arms. Similar to the analysis of OS, patients in the placebo arm with ≥ 3% BWL had the shortest median PFS (1.7 months) compared with the other BWL subgroups (ranging from 1.9 to 2.1 months).

The 451 patients included in these efficacy analyses also included patients with ascites. To eliminate patients for whom weight variation could be impacted by ascites, OS was analyzed after excluding patients with ascites. These analyses showed similar results (median OS ranging from 6.4 to 7.1 months for patients with < 3% BWL, and 2.7 to 4.6 months for patients with ≥ 3% BWL), as reported above. Among patients without ascites, 26 patients experienced any weight gain (3% or higher).

Comparisons of efficacy between the FTD/TPI and placebo treatment arms within the subgroups were confounded by the imbalance of weight loss between arms: patients who received placebo were more likely to have weight loss during the first treatment cycle (odds ratio 0.55; 52/95) than those who received FTD/TPI (odds ratio 0.36; 80/224). With this caveat, the treatment effect size was markedly higher in the ≥ 3% BWL subgroup (OS HR for FTD/TPI vs placebo was 0.40 [95% CI 0.26–0.60]; PFS HR was 0.36 [95% CI 0.24–0.55]) in comparison with the total (non-selected) population of 507 patients (OS HR for FTD/TPI vs placebo: 0.69 [95% CI, 0.56–0.85]; PFS HR: 0.57 [95% CI, 0.47–0.70]) [10].

### Exposure and safety

An analysis of FTD/TPI exposure in the < 3% and ≥ 3% BWL subgroups showed a similar dose intensity of FTD/TPI, but a slightly shorter mean duration of treatment in patients with ≥ 3% BWL (11.7 weeks and median of 2 cycles compared with 13.7 weeks and a median of 2.5 cycles in patients with < 3% BWL; Table S2). Among patients who received placebo, mean duration of treatment was shorter in patients with ≥ 3% BWL (4.9 weeks) than in patients with < 3% BWL (9.6 weeks).

Among patients who received FTD/TPI, the overall incidences of AEs and grade ≥ 3 events, as well as treatment-related AEs and grade ≥ 3 AEs were similar between the 2 BWL subgroups (Table 3). The frequencies of grade ≥ 3 hematologic AEs were mostly consistent across the subgroups among FTD/TPI-treated patients, except for anemia, which was more frequent in the ≥ 3% BWL subgroup (28% vs 17% in the < 3% group).

**Table 3:** AEs of any cause in patients with < 3% or ≥ 3% BWL

*n* (%)	Patients with < 3% BWL				Patients ≥ 3% BWL			
	FTD/TPI (*n* = 224)		Placebo (*n* = 95)		FTD/TPI (*n* = 80)		Placebo (*n* = 52)	
	Any grade	Grade ≥ 3	Any grade	Grade ≥ 3	Any grade	Grade ≥ 3	Any grade	Grade ≥ 3^b^
Summary of AEs								
Any AE	217 (97)	173 (77)	85 (89)	43 (45)	80 (100)	66 (82)	52 (100)	35 (67)
Treatment-related AEs	186 (83)	112 (50)	58 (61)	9 (9)	68 (85)	47 (59)	30 (58)	12 (23)
Serious AEs	85 (38)	78 (35)	23 (24)	22 (23)	34 (42)	31 (39)	28 (54)	26 (50)
Treatment-related serious AEs	21 (9)	16 (7)	2 (2)	2 (2)	10 (12)	9 (11)	3 (6)	3 (6)
AEs leading to treatment discontinuation	21 (9)	16 (7)	7 (7)	5 (5)	8 (10)	8 (10)	11 (21)	9 (17)
AEs leading to dose modification	126 (56)	91 (41)	14 (15)	11 (12)	53 (66)	43 (54)	17 (33)	12 (23)
Most common AEs (≥ 10% incidence in any subgroup)								
Hematologic								
Neutropenia^a^	128 (57)	80 (36)	7 (7)	0	43 (54)	30 (38)	0	0
Anemia^b^	101 (45)	38 (17)	20 (21)	5 (5)	40 (50)	22 (28)	9 (17)	7 (13)
Leukopenia^c^	60 (27)	25 (11)	3 (3)	0	14 (18)	4 (5)	0	0
Thrombocytopenia^d^	40 (18)	6 (3)	6 (6)	0	16 (20)	4 (5)	1 (2)	0
Gastrointestinal								
Nausea	76 (34)	5 (2)	28 (29)	3 (3)	36 (45)	2 (2)	21 (40)	2(4)
Diarrhea	53 (24)	4 (2)	10 (11)	0	17 (21)	2 (2)	13 (25)	2 (4)
Vomiting	56 (25)	7 (3)	16 (17)	0	17 (21)	1 (1)	16 (31)	2 (4)
Abdominal pain	37 (17)	8 (4)	17 (18)	7 (7)	14 (18)	4 (5)	12 (23)	6 (12)
Constipation	27 (12)	2 (1)	17 (18)	3 (3)	17 (21)	2 (3)	13 (25)	2 (4)
Upper abdominal pain	15 (7)	0	11 (12)	1 (1)	7 (9)	1 (1)	4 (8)	1 (2)
Dysphagia	10 (4)	4 (2)	3 (3)	2 (2)	8 (10)	1 (1)	4 (8)	1 (2)
Ascites	9 (4)	5 (2)	7 (7)	5 (5)	6 (8)	4 (5)	8 (15)	5 (10)
Other								
Decreased appetite	65 (29)	12 (5)	25 (26)	5 (5)	43 (54)	13 (16)	22 (42)	2 (4)
Fatigue	61 (27)	12 (5)	24 (25)	5 (5)	21 (26)	6 (8)	9 (17)	4 (8)
Asthenia	41 (18)	11 (5)	22 (23)	7 (7)	20 (25)	3 (4)	14 (27)	3 (6)
Blood alkaline phosphatase increased	22 (10)	6 (3)	9 (9)	3 (3)	8 (10)	3 (4)	5 (10)	2 (4)
Back pain	15 (7)	0	8 (8)	2 (2)	9 (11)	2 (2)	3 (6)	2 (4)
Pyrexia	16 (7)	0	2 (2)	0	8 (10)	0	3 (6)	0
AST increased	15 (7)	5 (2)	6 (6)	0	6 (8)	0	6 (12)	3 (6)
Dyspnea	14 (6)	3 (1)	8 (8)	2 (2)	8 (10)	2 (2)	5 (10)	2 (4)
General physical health deterioration	14 (6)	13 (6)	2 (2)	1 (1)	7 (9)	7 (9)	11 (21)	10 (19)
Hypoalbuminemia	11 (5)	1 (< 1)	2 (2)	0	7 (9)	1 (1)	6 (12)	1 (2)
Weight decreased	8 (4)	0	2 (2)	0	10 (12)	0	10 (19)	0
Peripheral edema	10 (4)	0	7 (7)	1 (1)	7 (9)	0	5 (10)	0
Insomnia	7 (3)	0	5 (5)	0	4 (5)	0	5 (10)	0
Hyponatremia	3 (1)	3 (1)	2 (2)	2 (2)	2 (2)	1 (1)	5 (10)	4 (8)

*AE* adverse event. *AST* aspartate aminotransferase. *BWL* body weight loss. *FTD/TPI* trifluridine/tipiracil

^a^Neutropenia and/or decreased neutrophil count

^b^Anemia and/or decreased hemoglobin level

^c^Leukopenia and/or decreased white blood cell count

^d^Thrombocytopenia and/or decreased platelet count

Among patients receiving placebo, a trend toward higher rates of grade ≥ 3 AEs, including treatment-related grade ≥ 3 AEs, was observed in patients with ≥ 3% BWL compared to those with < 3% BWL. Rates of grade ≥ 3 anemia were numerically higher in placebo-treated patients with ≥ 3% BWL than those with < 3% BWL (13% vs 5%) and appeared to be related to treatment (Table 3). While the frequency of gastrointestinal AEs trended higher in patients with ≥ 3% BWL, these variations were largely not attributed to treatment. Similarly, general physical health deterioration and hypoalbuminemia trended higher in the ≥ 3% BWL subgroup (21% and 12% of patients, respectively, compared with 2% each in the < 3% BWL subgroup); however, these events were unrelated to treatment.

Across both treatment arms, patients with ≥ 3% BWL experienced higher rates of decreased appetite (54%, FTD/TPI; 42%, placebo) than those with < 3% BWL (29%, FTD/TPI; 26%, placebo).

Overall rates of serious AEs of any cause were higher among patients in the ≥ 3% BWL subgroup than in the < 3% BWL subgroup, with the largest difference noted in patients treated with placebo (54% vs 24%; Table 3). Most serious AEs were, however, not related to treatment: across the < 3% and ≥ 3% subgroups, 9% and 12% in the FTD/TPI arm, and 2% and 6% in the placebo arm, respectively, experienced treatment-related serious AEs.

In both subgroups, AEs were managed by dosing modifications (Table 3); the proportions of FTD/TPI-treated patients who had dosing modifications due to AEs were 56% and 66% in the < 3% and ≥ 3% subgroups, respectively. The rates of permanent treatment discontinuations due to AEs were highest among those with ≥ 3% BWL receiving placebo (23%). In comparison, discontinuation rates due to AEs were 7% among placebo-treated patients with < 3% BWL, and 9% and 10%, respectively, among FTD/TPI-treated patients with < 3% and ≥ 3% BWL (Table 3).

## Discussion

The results of these analyses in TAGS indicated that ≥ 3% BWL at the end of treatment cycle 1 was associated with unfavorable survival outcomes in patients with mGC/GEJC treated in the third- or later-line setting. The negative association of early BWL with OS was observed in both FTD/TPI and placebo treatment groups. Univariate analysis showed that BWL was prognostic for OS, and multivariate analyses confirmed BWL to be both a prognostic (*p* < 0.0001) and predictive factor (interaction *p* = 0.0003) for OS. Similar findings were noted for PFS outcomes. While safety outcomes among FTD/TPI-treated patients did not appreciably vary based on early BWL, placebo-treated patients with ≥ 3% BWL experienced higher incidences of grade ≥ 3 events and treatment-related discontinuations than the other subgroups.

While mGC is associated with a poor prognosis [4], clinical outcomes in the first-, second- and third-line settings are improving as treatment options have expanded [6, 7, 10, 24–28]. This shift in the treatment landscape makes a more detailed understanding of prognostic factors in such settings a more relevant aspect to clinical decision-making. The post hoc analysis of early weight loss in patients from 3 large, phase III randomized controlled trials of ramucirumab (REGARD, RAINBOW and RAINFALL) showed the importance of minimizing early weight loss in the first- and second-line setting [18]. Data from a total of 1464 patients were included, with patients categorized by weight loss of ≥ 3% or < 3% of body weight during the first cycle of treatment. In that analysis, ≥ 3% BWL within the first 3–4 weeks of systemic treatment had a consistent negative prognostic effect on OS in patients with advanced gastric or gastroesophageal junction adenocarcinoma. Our analysis from the TAGS trial expands these findings to a population of heavily pretreated patients, over 50% of whom had received 3 or more prior treatments for mGC/GEJC. Preliminary analyses indicated that the exclusion of patients with ascites did not affect these conclusions, indicating that the weight gain or loss in cycle 1 was not due to water retention or diuresis associated with ascites. These results are consistent with prior data and indicate that a 3% BWL in the first cycle of treatment serves as a robust prognostic factor for survival in patients treated in the third- or later-line setting.

Given the importance of keeping patients well across lines of therapy for mGC, safety and tolerability are relevant considerations for individualized patient care. Although correlations of OS and PFS with early BWL have been previously studied [18, 23], the safety of systemic therapy has not been evaluated in the context of BWL. In these analyses, early BWL did not appear to correlate with increases in FTD/TPI-related toxicity, although higher frequencies of decreased appetite correlated with higher BWL, as may be expected. However, placebo-treated patients with ≥ 3% BWL experienced markedly higher toxicity, including higher frequencies of grade ≥ 3 AEs and AE-related discontinuations than those with < 3% BWL. These increases in AE incidences were not related to treatment, for the most part, and were more likely a consequence of the worse prognosis in placebo-treated patients who experienced greater weight reduction. Together with the effect of early BWL on survival in placebo-treated patients with BWL ≥ 3% (who had notably shorter survival than those with BWL < 3%), these data indicate that early BWL had the most pronounced impact on outcomes in the placebo group, both from an efficacy and a toxicity perspective.

These results also imply that FTD/TPI treatment may help mitigate both weight loss and the negative effects of weight loss. Patients receiving FTD/TPI were less likely to experience weight loss during the first treatment cycle than those receiving placebo. Among patients who experienced ≥ 3% BWL, those receiving FTD/TPI stayed longer on treatment than those receiving placebo (11.7 vs 4.9 weeks). Finally, the treatment effect size was more pronounced in those with ≥ 3% BWL (OS HR, 0.40 for FTD/TPI vs placebo in the ≥ 3% BWL subgroup compared with an OS HR of 0.69 for FTD/TPI vs placebo among all patients). Although these results must be interpreted with caution, given the imbalances between the treatment arms, they nevertheless indicate the potential benefits of FTD/TPI treatment even in patients at risk for weight loss.

The detection of early BWL, even as low as 3%, could also have implications for prognosis. Because the weight cutoff is low, it may present an opportunity for intervention to regain BWL in subsequent cycles, with the potential to improve outcomes in these patients. Unfortunately, analyses of weight gain/loss in subsequent cycles could not be carried out for TAGS because of high treatment discontinuation rates following cycle 2. However, such analyses are relevant for the future to examine whether interventions for early BWL can impact survival in patients with mGC/GEJC.

It may be pertinent to examine if in converse, patients with weight gain experience any additional benefit from treatment. However, we were limited by extremely small numbers of patients (< 6% of 451 patients) to analyze this further in TAGS. A consideration for evaluating the impact of weight gain is the importance of muscle gain and muscle preservation. Sarcopenia, or decreased skeletal muscle mass, has been associated with poor survival in patients with GC and other cancers [25, 29–33]. In addition to baseline and preoperative sarcopenia being predictive of poor prognosis [30, 31], a retrospective study of 118 patients found that muscle loss during chemotherapy, defined as a ≥ 10% reduction in skeletal muscle index, negatively affected survival in patients with mGC [29]. Sarcopenia was not assessed in the TAGS trial but understanding the impact of baseline sarcopenia and muscle loss during treatment will provide further insight into the relationship between BWL and treatment outcomes.

### Limitations

A strength of this analysis was the relatively large patient population of the TAGS trial. One key limitation was the exploratory post hoc nature of this analysis, which was not powered to investigate differences in efficacy and safety between the BWL subgroups. Imbalances in weight loss between the treatment arms precluded comparisons between treatment arms in this analysis. Imbalances in other baseline characteristics, including ECOG PS between the BWL subgroups may have had an impact on tolerability. Finally, longitudinal weight data over subsequent cycles could not be measured, because many patients, particularly those in the placebo group, discontinued after the end of the first cycle of treatment. The mechanisms underlying poor survival outcomes in patients with ≥ 3% BWL remain unclear, and in this regard, an examination of the link between BWL and inflammatory indices such as C-reactive protein or with baseline neutrophil to lymphocyte ratio will be helpful for future analyses [34].

## Conclusions

In conclusion, this analysis showed that BWL at the end of cycle 1 of treatment was associated with unfavorable survival outcomes in patients with mGC/GEJC in the TAGS trial, regardless of FTD/TPI or placebo treatment. As observed in earlier settings [17, 18, 21, 23], early BWL was a strong negative prognostic marker for OS in the third- or later-line setting for mGC/GEJC.

## Supplementary Information

Below is the link to the electronic supplementary material.

Supplementary file1 (DOCX 16 KB)

## Data Availability

Data generated or analyzed during this study are on file with Taiho Oncology, Inc., and Taiho Pharmaceuticals Co., Ltd., and are not publicly available. Inquiries about data access should be sent to th-datasharing@taiho.co.jp**.**
