# Supplementary material for: Body weight loss as a prognostic and predictive factor in previously treated patients with metastatic gastric cancer: post hoc analyses of the randomized phase III TAGS trial
Source: Gastric Cancer. 2023 Apr 28;26(4):626–37. doi: 10.1007/s10120-023-01393-2 (PMC10284730; doi:10.1007/s10120-023-01393-2)
Supplement: Supplementary file 1 — Supplementary file1 (DOCX 16 KB) [file 10120_2023_1393_MOESM1_ESM.docx]

**Supplementary Appendix**

Body Weight Loss as a Prognostic and Predictive Factor in Previously Treated Patients with Metastatic Gastric Cancer: *Post Hoc* Analyses of the Randomized Phase 3 TAGS Trial

Michele Ghidini,^1^ Howard Hochster,^2^ Toshihiko Doi,^3^ Eric Van Cutsem,^4^ Lukas Makris,^5^ Karim A. Benhadji,^6^ Wasat Mansoor^7^

# Table S1: Patient disposition in patients with <3% or ≥3% BWL

|  | **Patients with <3% BWL** | | | **Patients ≥3% BWL** | | |
| --- | --- | --- | --- | --- | --- | --- |
|  | **FTD/TPI (n=224)** | **Placebo (n=95)** | **FTD/TPI (n=80)** | | **Placebo (n=52)** |  |
| **Ongoing study treatment, n (%)** | 17 (8) | 3 (3) | 2 (3) | | 0 |  |
| **Discontinued study treatment,** **n (%)**  AEs  Clinical progression  Radiological progression  Patient withdrew consent  Physician’s decision  Pregnancy  Death  Other  Protocol violation | 207 (92)  17 (8)  31 (14)  138 (62)  6 (3)  9 (4)  0  5 (2)  1 (<1)  1 (<1) | 92 (97)  3 (3)  13 (14)  72 (76)  2 (2)  2 (2)  0  0  0  0 | 78 (98)  6 (8)  13 (16)  51 (64)  5 (6)  2 (2)  0  1 (1)  0  0 | | 52 (100)  3 (6)  12 (23)  35 (67)  0  1 (2)  0  1 (2)  0  0 |  |

*AE* adverse event. *BWL* body weight loss. *FTD/TPI* trifluridine/tipiracil.

#

# Table S2. Treatment exposure in patients with <3% or ≥3% BWL

|  | **Patients With < 3% BWL** | | **Patients With ≥ 3% BWL** | |
| --- | --- | --- | --- | --- |
|  | **FTD/TPI**  **(n=224)** | **Placebo**  **(n=95)** | **FTD/TPI**  **(n=80)** | **Placebo**  **(n=52)** |
| Mean (SD) dose intensity, mg/m^2^/week | 148.8 (24.1) | 156.4 (22.7) | 146.5 (27.1) | 154.6 (27.6) |
| Mean (SD) dose intensity (ratio to planned) | 0.9 (0.1) | 0.9 (0.1) | 0.8 (0.2) | 0.9 (0.2) |
| Median (range) cycles initiated per patient | 2.5 (1−14) | 2.0 (1−16) | 2.0 (1−12) | 2.0 (1−3) |
| Mean (SD) treatment duration, weeks | 13.7 (11.8) | 9.6 (9.5) | 11.7 (10.5) | 4.9 (2.5) |

*BWL* body weight loss. *FTD/TPI* trifluridine/tipiracil. *SD* standard deviation.
